# Roof Gardens for the People

**Published:** 1897-08

**Authors:** 


### ROOF GARDENS FOR THE PEOPLE.

NEW YORK'S new recreation pier at the foot of Third street, East River, has proven a boon to a class of humanity that finds it difficult to get a puff of fresh air even in the daytime, to say nothing about nights in their overcrowded tenements.

Whoever proposed to utilise the pier-houses by adding roof-gardens to their tops is a philanthropist in the fullest sense of the word. The new pier referred to is a well-built two-story fire-proof structure, with iron stairways leading from the street to the recreation floor, which latter has an arched iron roof with open sides between the girders and is illuminated at night with arc lights.

On hot afternoons the place is crowded, while at night there is literally a crush. At the entrance perambulators are left, which creates a blockade of empty baby carriages. Generally speaking, a band plays at night as well as upon several afternoons during the week. Quick music sets the youngsters to dancing and popular airs start them all a-singing ; so that often the singing, shouting and whistling are almost deafening and pandemonium seems let loose. One can scarcely hear the band play half the length of the pier, yet no one is disturbed by the noise, for it means health and happiness to those sadly in need of both.

It takes very little to make these people happy : a penny's worth of ice cream being sufficient to delight the hearts of two children and those with more delicate stomachs may obtain sterilised milk for a small outlay, while for the robust there is candy and open-face pies galore—all for a few pennies.

The health-preserving powers of these piers is beyond estimation, and New York has set an example that is worth emulating. Here the poor, without money and without price, can breathe pure air, listen to good music and spend evenings in innocent amusement that is health-promoting.

Several uniformed employees of the dock department and a few policemen keep order on the floor, while two matrons, in black with white aprons, collars and cuffs, look after the comfort of women and babies. Municipalities are fast learning that it is economy to prevent sickness by affording opportunities for the poor to work and to provide them with innocent recreation as well as free baths.

